# Hippocampus‐prefrontal cortex inputs modulate spatial learning and memory in a mouse model of sepsis induced by cecal ligation puncture

**DOI:** 10.1111/cns.14013

**Published:** 2022-11-15

**Authors:** Cheng‐Long Ge, Wei Chen, Li‐Na Zhang, Yu‐Hang Ai, Yu Zou, Qian‐Yi Peng

**Affiliations:** ^1^ Department of Critical Care Medicine Xiangya Hospital, Central South University Changsha Hunan Province China; ^2^ National Clinical Research Center for Geriatric Disorders Changsha Hunan Province China; ^3^ Hunan Provincial Clinical Research Center for Critical Care Medicine Changsha Hunan Province China; ^4^ Department of Anesthesia Xiangya Hospital, Central South University Changsha Hunan Province China

**Keywords:** cecal ligation puncture, cognitive dysfunction, hippocampus, prefrontal cortex, sepsis‐associated encephalopathy

## Abstract

**Aims:**

Sepsis‐associated encephalopathy (SAE) often leads to cognitive impairments. However, the pathophysiology of SAE is complex and unclear. Here, we investigated the role of hippocampus (HPC)‐prefrontal cortex (PFC) in cognitive dysfunction in sepsis induced by cecal ligation puncture (CLP) in mice.

**Methods:**

The neural projections from the HPC to PFC were first identified via retrograde tracing and viral expression. Chemogenetic activation of the HPC‐PFC pathway was shown via immunofluorescent staining of c‐Fos‐positive neurons in PFC. Morris Water Maze (MWM) and Barnes maze (BM) were used to evaluate cognitive function. Western blotting analysis was used to determine the expression of glutamate receptors and related molecules in PFC and HPC.

**Results:**

Chemogenetic activation of the HPC‐PFC pathway enhanced cognitive dysfunction in CLP‐induced septic mice. Glutamate receptors mediated the effects of HPC‐PFC pathway activation in CLP mice. The activation of the HPC‐PFC pathway resulted in significantly increased levels of NMDAR, AMPAR, and downstream signaling molecules including CaMKIIa, pCREB, and BDNF in PFC. However, inhibition of glutamate receptors using 2,3‐dihydroxy‐6‐nitro‐7‐sulphamoyl‐benzo (F)quinoxaline (NBQX), which is an α‐amino‐3‐hydroxy‐5‐methyl‐4‐isoxazolepropionic acid receptor (AMPAR inhibitor), or D‐2‐amino‐5‐phosphonopentanoate (D‐AP5), which is an NMDA receptor antagonist abolished this increase.

**Conclusion:**

Our study reveals the important role of the HPC‐PFC pathway in improving cognitive dysfunction in a mouse model of CLP sepsis and provides a novel pathogenetic mechanism for SAE.

## Graphical abstract

The proposed mechanism of sepsis‐associated encephalopathy. HPC‐PFC pathway plays an important role in cognitive dysfunction in sepsis‐associated encephalopathy (SAE). Specifically, CaMKII/CREB/BDNF pathway in the glutamate receptor‐mediated downstream signaling appears to be an important molecular mechanism linking the HPC‐mPFC pathway with impairing spatial memory in SAE.

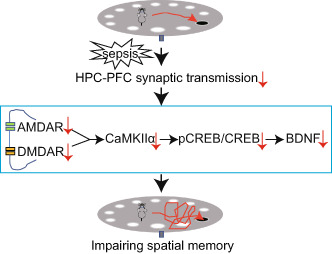

## 1 INTRODUCTION

Sepsis is a systemic inflammatory response triggered by severe local infection, which can lead to multiorgan dysfunction and death. Approximately more than 19 million people develop sepsis.^1^ The central nervous system (CNS) is often affected in the early stages of sepsis, which leads to sepsis‐associated encephalopathy (SAE) accounting in approximately 49.6% of all patients diagnosed with sepsis.^2^ However, the pathogenetic mechanisms associated with cognitive dysfunction in SAE are poorly understood.

Studies involving mice show that synchronized neuronal activity occurs in the medial prefrontal cortex (mPFC) and the ventral hippocampus (vHPC) during spatial memory, the intensity of synchronization is associated with behavioral performance.^3^, ^4^, ^5^ The HPC‐PFC pathway plays an important cognitive role, including concentration, decision making, and short‐term and long‐term memory.^6^, ^7^ The HPC‐PFC pathway has been associated with neuropathic pain,^8^ depression,^9^, ^10^ and Alzheimer's disease (AD).^11^, ^12^ In addition, glutamate neurotransmission plays a critical role in the HPC‐PFC pathway, as well as in cognition and memory.^13^

Cognitive dysfunction is a common manifestation of SAE. Studies suggest that structural abnormalities associated with hippocampus or prefrontal cortex^14^, ^15^ contribute to cognitive impairment in SAE. Our previous work indicated that chemogenetic activation of the HPC‐PFC pathway improved short‐term learning and memory dysfunction in acute brain injury following lipopolysaccharide (LPS)‐induced sepsis. Here, we further investigated the role of the HPC–PFC pathway in regulating spatial learning and memory of mice starting a week after cecal ligation and puncture (CLP)‐induced sepsis until 2 weeks. We confirmed neural projections from the HPC to the PFC using fluorescent tracers and viruses. We analyzed the role of chemogenetic activation of the HPC‐PFC pathway in the performance of mice on Morris Water Maze (MWM) and Barnes maze (BM). We also explored the molecular mechanism of the HPC‐PFC pathway in SAE.

## 2 MATERIALS AND METHODS

### 2.1 Animals

One hundred and sixty male C57BL/6 mice, each weighing between 18 g and 22 g initially, and aged 6–8 weeks, were purchased from Hunan SJA Laboratory Animal Co., Ltd. (Changsha, China). All procedures were carried out according to the Guideline for the Care and Use of Laboratory Animals (Bethesda, MD, United States). All animal experiments were approved by the Animal Care and Use Committee of Central South University (No. 2019sydw0240). Mice were housed in groups of 4–5 animals per cage with access to food and water ad libitum, and exposed to a 12/12 h light/dark cycle. The experiments were started after at least 2 weeks of acclimatization.

### 2.2 Experimental design

The experiment was divided into two segments. The first segment explored the role of the HPC‐PFC pathway in cognitive dysfunction of CLP‐induced sepsis. The second segment investigated the potential underlying mechanism and the signaling pathway involved.

**FIGURE 1:**
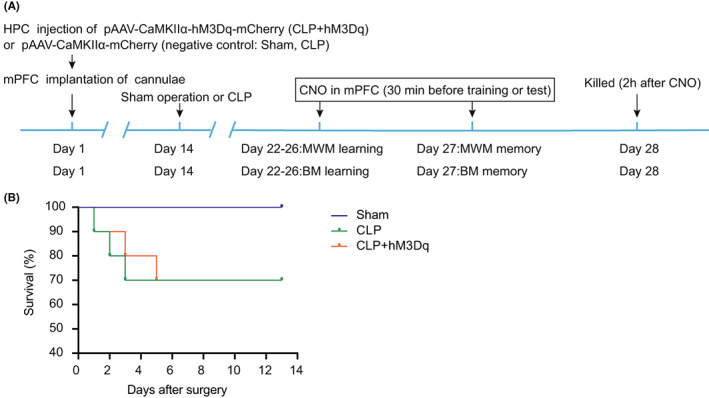
Flow chart of experimental design and effects of chemogenetic activation of the HPC‐mPFC pathway on the survival rate of mice post‐CLP. Chemogenetic activation of the HPC‐mPFC pathway did not affect the survival rate of mice after CLP (*n* = 10 per group). The survival rate of mice in the Sham operation group was 100%. The overall survival rate of mice in both CLP and CLP + hM3Dq groups was 60%. vHPC, ventral hippocampus; mPFC, medial prefrontal cortex; CNO, clozapine‐N‐oxide; MWM, Morris Water Maze; BM, Barnes maze; CLP, cecal ligation and puncture.

The experimental design of the first segment is summarized in Figure 1. Mice were randomly divided into three groups: Sham (*n* = 20), CLP (*n* = 20), and CLP + hM3Dq (*n* = 20). Purified adeno‐associated viral (AAV) vector expressing the CaMKII*α*‐hM3Dq‐mCherry receptor (pAAV‐CaMKII*α*‐hM3Dq‐mCherry) or pAAV‐CaMKII*α*‐mCherry alone was injected into vHPC on day 1 bilaterally. To facilitate clozapine‐N‐oxide (CNO) injection, mice were bilaterally implanted with a pair of guide cannulas into mPFC concurrently. CLP or Sham‐operated model was induced after 2 weeks. The behavioral tests including MWM and BM were performed on post‐surgical day 7. The survival rate of mice was monitored for 14 days following CLP.

The remaining mice (*n* = 100) were used in glutamate receptor (GluR) antagonist experiments (segment 2, Figure 5). The methods of virus injection, cannula implantation, and modeling were described previously.^16^, ^17^ Glutamate receptor antagonist or saline was injected bilaterally using the guide cannulas 30 min prior to CNO injection (Figure 5A). MWM and BM were used to assess spatial learning and memory in animals as described before. Mice were killed after completion of the behavioral tests for immunofluorescence testing and western blotting analysis of PFC and HPC.

### 2.3 Cecal ligation and puncture model

Mice were subjected to CLP to induce SAE as previously described.^18^ Briefly, mice were anesthetized via intraperitoneal injection of pentobarbital sodium (60 mg/kg), followed by a ventral midline incision (1 cm). The cecum was isolated and ligated using 3.0 silk approximately 1 cm from the distal end and punctured with a 21‐gauge needle. The stool was then expelled from the puncture wound by gently squeezing the cecum. Finally, the cecum was gently returned to the abdominal cavity and the abdomen was sutured. The mice were immediately resuscitated with normal saline (20 ml/kg) subcutaneously after surgery. Mice in the Sham group were subjected to the same surgery as described above but without cecal ligation or puncture. The survival rate was monitored for 14 days following CLP (Figure 1B).

### 2.4 Drugs and virus

Fluoro‐Gold (4% in 0.9% saline, Fluorochrome, LLC); clozapine‐N‐oxide (CNO; 1 mM dissolved in 0.9% saline, MedChemExpress), NBQX, or 2,3‐dihydroxy‐6‐nitro‐7‐sulphamoyl‐benzo (F)quinoxaline, which is an α‐amino‐3‐hydroxy‐5‐methyl‐4‐isoxazolepropionic acid receptor (AMPAR inhibitor); D‐AP5 or D‐2‐amino‐5‐phosphonopentanoate, which is an NMDA receptor antagonist (1 μg/μl dissolved in 0.9% saline; MedChemExpress), and KBQX (10 nmol/ml dissolved in 0.9% saline, AMDAR inhibitor; MedChemExpress) were used.

The pAAV‐CaMKII*α*‐hM3Dq‐mCherry and pAAV‐CaMKII*α*‐mCherry used in the present study were verified and purchased from OBiO Technology (Shanghai, China) Corp., Ltd.

### 2.5 Retrograde tracing

**FIGURE 2:**
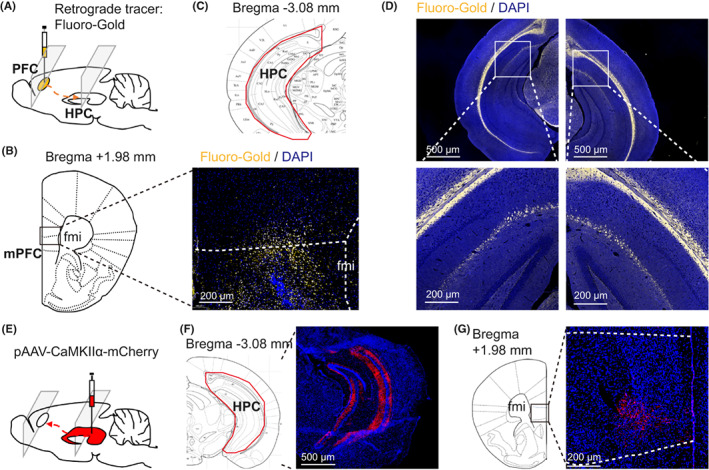
Hippocampus sends neural projections to the medial prefrontal cortex. (a‐d) Retrograde tracing: (A) microinjection of retrograde fluorescent tracer Fluoro‐Gold into the medial prefrontal cortex (mPFC); (B) a representative image showing the presence of Fluoro‐Gold in the mPFC; (C) representative figures showing the location of the ventral hippocampus (vHPC); (D) Fluoro‐Gold microinjected into the mPFC cells detected in the vHPC after 4 days. (E–G) Anterograde tracing: (E) Microinjection of pAAV‐CaMKII*α*‐mCherry into the vHPC; (F) after 4 weeks, a representative image showing the expression of mCherry in the vHPC; (G) mCherry was probed in the mPFC after viral microinjection into the vHPC.

To validate the construction of neurons projecting to the mPFC, an additional 5 mice were anesthetized via 2% isoflurane inhalation and fixed on a stereotaxic instrument, where the retrograde tracer Fluoro‐Gold (0.5 μl/side) was injected bilaterally into the mPFC (anteroposterior (AP) + 1.8 mm, mediolateral (ML) ± 0.4 mm, dorsoventral (DV) ‐1.4 mm)^7^ using microinjection needles (33 G). Approximately 4–7 days after the injection, the mice were anesthetized and killed by decapitation. The whole brain tissue was removed and frozen immediately. The frozen brain sections (10 μm) were treated with Fluoro‐Gold for retrograde tracing in the mPFC and vHPC (AP ‐3.1 mm, ML ± 3.0 mm, DV ‐3.9 mm)^7^ (Figure 2A–D).

### 2.6 Stereotaxic microinjection and cannula implantation

Mice were deeply anesthetized with sodium pentobarbital (60 mg/kg, i.p.). The head was fixed on a stereotaxic instrument (RWD Life Sciences, Shenzhen, China), followed by injection with pAAV‐CaMKII*α*‐hM3Dq‐mCherry or pAAV‐CaMKII*α*‐mCherry (negative control) bilaterally into the vHPC (0.5 μl/side) with Hamilton syringes (33 G, Hamilton Reno, NV) at a rate of 0.1 μl/min. The needles were left for another 5 min to facilitate adequate diffusion.

Bilateral cannulas (OD 0.48 mm, ID 0.34 mm) were implanted in the mPFC (AP 1.80, ML ± 0.40, DV ‐1.40).^7^ Approximately 4 weeks after the AAV injection, CNO (0.5 μl/side) or GluR antagonist was infused bilaterally into the cannulas. The needles were left in place for more than 5 min to facilitate drug diffusion.

### 2.7 Behavioral tests

All behavioral tests were conducted between 09:00 and 17:00, and each mouse was subjected to a single behavioral test only.

#### 2.7.1 Morris Water Maze

Morris Water Maze (MWM) was used to assess spatial learning and memory abilities of the mice. The MWM consisted of a circular pool (1.2 m in diameter) filled with water (22 ± 2°C) and a platform (10 cm in diameter) submerged 1 cm below the surface of water, as described previously.^19^ A white food color was added to the water to make it opaque. The spatial learning task was conducted by training the mice to detect the platform for 5 consecutive days during 4 trials each day, with each trial lasting 60 s. In the spatial memory task, the platform was removed, and each mouse was placed gently into the pool at the same location and allowed to explore freely for 2 min. The escape latency during spatial learning, and the swimming paths, the primary latency to the target site, the swimming speeds, the frequency of platform crossings, and the time spent in the target quadrant in the spatial memory task were recorded and analyzed using Smart 3.0 software.

#### 2.7.2 Barnes maze

Barnes maze (BM) consisted of a round platform measuring 105 cm in height and 92 cm in diameter, with 20 holes (1 target hole and 19 empty holes). Before each trial, the mice were placed in the center of the platform. During the learning phase, the mice were trained to find the target hole within 3 min in each trail for 5 consecutive days, with 3 trials daily. After each trial, the maze was cleaned with 70% alcohol to reduce the odor cues. The escape box was removed on day 6, and the mice were allowed to freely explore the maze for 3 min. During the learning task, the latencies to the target hole were recorded. During the memory task, the search paths, primary latency to the target hole, percentage of correct pokes, and the total distance traveled were recorded and analyzed.

### 2.8 Immunofluorescent staining and image analysis

Immunofluorescent staining was performed as previously reported.^16^ Mice were anesthetized with 2% isoflurane and perfused with 0.9% saline, followed by 4% paraformaldehyde. The whole brains were rapidly isolated and fixed in 4% paraformaldehyde overnight and sliced into 10 μm‐thick frozen sections at the vHPC and mPFC levels. The frozen sections were washed 3 times for 10 min in phosphate‐buffered saline (PBS) and then incubated with blocking buffer (3% BSA in PBS, 0.1% Triton ×100) for 50 min. The sections were subsequently incubated at 4°C overnight with the following primary antibodies: anti‐mCherry (1:500, ab183628, Abcam) and anti‐c‐Fos (1:500, ab208942, Abcam). On the following day, the sections were washed 3 times with PBS and incubated with the corresponding secondary antibodies diluted in PBS (1:500) for 60 min. The secondary antibodies included Alexa Fluor 488 (ab150113, Abcam) and Alexa Fluor 594 (ab150080, Abcam). After washing 3 times in PBS, the sections were re‐stained with 4′, 6‐diamidino‐2‐phenylindole (DAPI) and analyzed under a fluorescence microscope. Image J software was used to count the number of c‐Fos‐positive cells. Fluoro‐Gold was directly detected and photographed with ultraviolet light (380 nm excitation wavelength, 420 nm absorption wavelength) under a fluorescence microscope.

### 2.9 Western blotting analysis

The hippocampus and prefrontal cortex were carefully isolated and stored at −80°C until use. The proteins were extracted with a total protein extraction kit (KeyGEN BioTECH, Cat. No. KGP902). The protein samples were separated via 8% SDS polyacrylamide gel electrophoresis (8% SDS‐PAGE) and then transferred to PVDF membranes. After blocking with a rapid blocking buffer for 20 min, the membranes were incubated with primary antibodies overnight at 4°C: CREB (1:1000), pCREB (1:500), BDNF (1:1000), NR2A (1:500), NR2B (1:500), NR1 (1:1000), GluA1 (1:1000), GluA2 (1:1000), CaMKII (1:1000), Actin (1:5000) and GAPDH (1:5000). These antibodies were purchased from Bioworld Technology (Bioworld, Louis Park, MN). The membranes were then detected with a secondary antibody HRP‐labeled goat anti‐rabbit IgG (1:1000, Cell Signaling Technology) and visualized via chemiluminescence. The protein bands were quantitatively analyzed using Image J software.

### 2.10 Statistical analyses

Statistical analysis was performed using GraphPad Prism 9.0 (GraphPad software, San Diego, CA). Data were presented as mean ± standard error of the mean. Following a D'Agostino‐Pearson's test, the normality of the distributions was determined, followed by multiple comparison of the groups via one‐way analysis of variance (ANOVA) and post hoc Tukey test. If the data were not normally distributed, the Kruskal‐Wallis test was used to compare 3 or more groups. Time series data were analyzed with repeated measures two‐way ANOVA. *p* < 0.05 was considered statistically significant.

## 3 RESULTS

### 3.1 Effects of HPC‐PFC pathway activation on 14‐day survival of mice after CLP

First, based on previous studies,^16^, ^20^ we determined the effective activated dose and initiation of CNO treatment for CLP‐induced sepsis. CNO (1 mM, 0.5 μl/side) was microinjected into the mPFC 30 min before each behavioral intervention, as shown in Figure 1A. The survival of mice was then monitored for 14 days. The survival rate of mice in the Sham group was 100%. The overall survival rate of mice in both CLP and CLP + hM3Dq groups was 60%. Activation of the HPC‐PFC signaling pathway did not affect the overall survival outcome (Figure 1B).

### 3.2 Identification and activation of the HPC‐PFC pathway

To internally validate the neural projections from the HPC to PFC, we first injected the retrograde fluorescent tracer Fluoro‐Gold into the mPFC of mice (Figure 2A, B). After 4 days of retrograde tracing, neurons in the HPC but not the adjacent brain areas were labeled with Fluoro‐Gold (Figure 2C, D). Anterograde tracing experiments were then performed to identify the projections arising from the HPC neurons to the PFC. We microinjected pAAV‐CaMKIIα‐mCherry into the HPC and evaluated the neural terminals in the mPFC (Figure 2E, F). Four weeks later, we detected a strong expression of mCherry (red) in the mPFC (Figure 2G).

**FIGURE 3:**
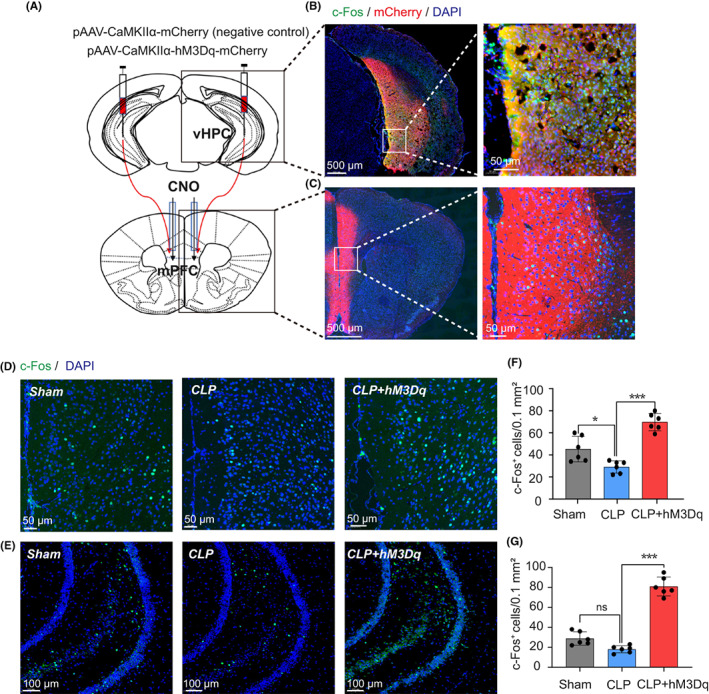
Chemogenetic activation of the HPC–PFC pathway. (A) Representative figures showing chemogenetic activation of the HPC–mPFC pathway. (B) A representative image showing the expression of hM3Dq‐mCherry in the vHPC. (C) A representative image showing c‐Fos and hM3Dq‐mCherry expression in the mPFC. (D, E) Representative images showing c‐Fos expression in the mPFC (D) and HPC (E) in different groups of mice. (F, G) Chemogenetic activation of the HPC–mPFC pathway increased the number of c‐Fos‐positive cells in the mPFC (F) and HPC (G) of mice with CLP‐induced sepsis, respectively. CLP‐induced sepsis decreased the number of c‐Fos‐positive cells in mPFC (F) but not in HPC (G). Data were presented as mean ± SEM. *N* = 6/group. *p < 0.05, ***p < 0.001.

To specifically activate the HPC‐PFC pathway, we used the chemical genetic method.^21^ We microinjected the pAAV‐CaMKIIα‐hM3Dq‐mCherry expressing excitatory designer receptors, which were exclusively activated by chemogenetic proteins (Designer Receptors Activated Only by Designer Drugs or DREADDs) such as hM3Dq, which is linked to Gαq signaling to trigger neurons, into the HPC, and then 4 weeks later, CNO (1 mM, 0.5 μl/side) was microinjected via cannula implanted into the mPFC (Figure 3A–C). The activation of the HPC‐PFC pathway by CNO was determined by analyzing the expression of c‐Fos (a marker of neuronal activation) in the mPFC and HPC (Figure 3D–G). Compared with the Sham group, mice treated with CLP carried a reduced number of c‐Fos‐positive neurons in the mPFC (Figure 3F), while no obvious difference was detected in the HPC (Figure 3G). The microinjection of CNO into the mPFC expressing hM3Dq increased the expression of c‐Fos both in mPFC (Figure 3F) and HPC (Figure 3G).

### 3.3 Chemogenetic activation of the HPC‐PFC pathway improved CLP‐induced cognitive dysfunction

**FIGURE 4:**
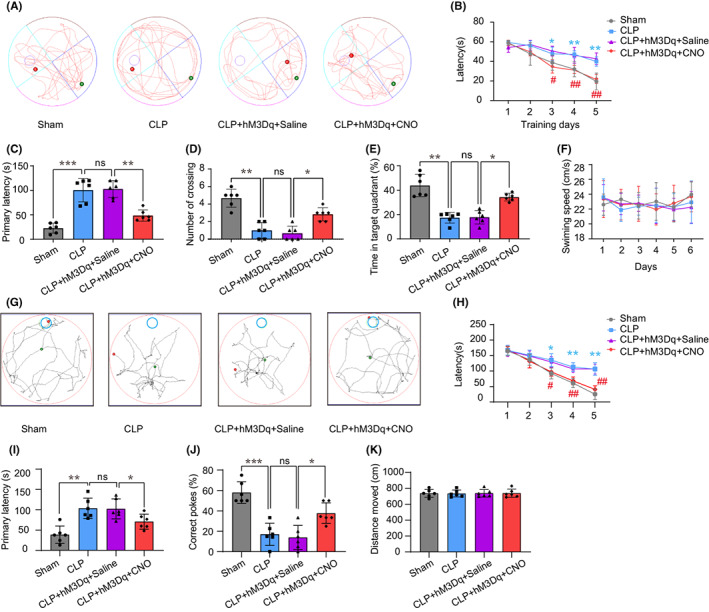
Activating HPC–PFC rescues CLP‐induced memory deficit. Spatial learning and memory were tested via Morris Water Maze (MWM) (A–F) and Barnes maze (BM) (G–K). (A) Representative swimming paths during probe trial in MWM. The blue dots represent the starting points and the red dots represent the final points. (B–E) MWM data show that the activation of HPC‐mPFC rescued CLP‐induced learning (B) and memory deficits (C–E). (F) The locomotion was not affected (*p* > 0.05). (G) Representative searching paths during the probe trial on BM. (H–J) BM data show that the activation of HPC–mPFC rescued CLP‐induced learning (H) and memory deficits (I, J). (K) The total distance traveled was not significantly different (*p* > 0.05), (*n* = 6/group), via repeated‐measures two‐way ANOVA.* CLP versus Sham; # CLP + hM3Dq + CNO versus CLP + hM3Dq + saline; *p < 0.05, **p < 0.01, ***p < 0.001, # *p* < 0.05, ## *p* < 0.01).

To investigate the effect of chemogenetic activation of the HPC‐PFC pathway on cognitive function of mice with CLP‐induced sepsis, we performed two behavioral tests: MWM and BM. As shown in Figure 4A, the representative swimming paths of mice crossed different quadrants after the platform in the MWM test was removed (day 6). Compared with the Sham group, the CLP group of mice showed fewer platform crossings and spent shorter duration in the target quadrant. CLP mice showed longer escape latencies than the Sham group during the learning task. The mice in the CLP + hM3Dq + CNO group spent less time searching for the hidden platform compared with mice in the CLP + hM3Dq + Saline group during the learning task (Figure 4B). During the memory test, the CLP mice showed longer escape latency, reduced frequency of crossing the area containing the platform location and spent less time in the target quadrant compared with the Sham group. The CLP + hM3Dq + CNO group showed shorter escape latency, a higher frequency of platform crossings, and longer duration in the target quadrant compared with mice in the CLP + hM3Dq + Saline group (Figure 4C–E). We found no differences in motor ability based on swimming speed (Figure 4F).

BM was used to test spatial memory. As shown in Figure 4G, the representative tracer movements indicate that the CLP group of mice spent longer than the Sham group exploring the target hole during the five consecutive training days. The findings were similar to those of MWM. Similarly, during the learning phase in the BM test, the CLP + hM3Dq + CNO group spent less time than the mice in the CLP + hM3Dq + Saline group exploring the target hole (Figure 4H). During the memory test, the CLP group showed longer primary latency and fewer correct pokes than the Sham group. The CLP + hM3Dq + CNO group also exhibited shorter primary latency and a higher number of correct pokes than the CLP mice (Figure 4I, J). No significant differences between the groups were detected in terms of distance traveled (Figure 4K).

These findings suggest that CLP‐induced sepsis results in impaired cognition and defective spatial memory. The behavioral data indicate that chemogenetic activation of the HPC‐PFC pathway improved CLP‐induced cognitive dysfunction.

### 3.4 Glutamate receptors mediated the effects of HPC‐PFC pathway activation in CLP mice

**FIGURE 5:**
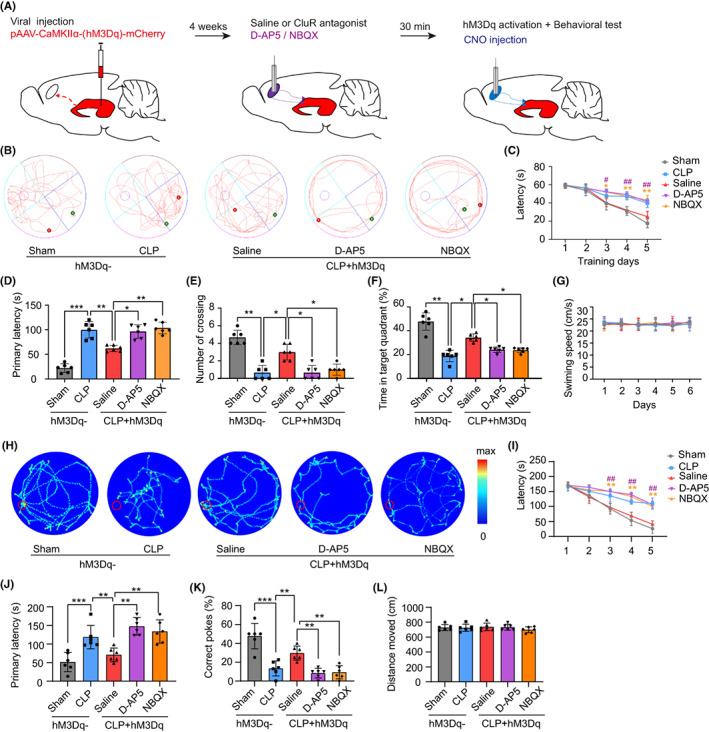
Glutamatergic HPC inputs to the PFC mediate changes in memory‐related behavior. (A) To selectively express hM3Dq in vHPC projection neurons, we injected bilaterally 0.5 μl of pAAV‐CaMKIIα‐hM3Dq‐mCherry into the vHPC (AP ‐3.1 mm, ML ± 3.0 mm, DV ‐3.9 mm) and implanted a pair of guide cannulas in mPFC (AP + 1.8 mm, ML ± 0.4 mm, DV ‐1.4 mm). Next, 0.5 μl of the glutamate receptor antagonist or saline was injected bilaterally via infusion cannulas 30 min prior to CNO injection. (B–M) Bilateral D‐AP5 or NBQX injection in the mPFC significantly prevented CNO‐induced memory consolidation in memory‐related behaviors in the Morris Water Maze (MWM) (B–G) and Barns maze (BM) tests (H–M) relative to saline trials. (B) Representative swimming paths during the probe trial in MWM (blue dots represent the starting points and red dots represent the final points). (C–F) MWM data show that the microinjection of intra‐mPFC GluR antagonists in mice expressing hM3Dq in vHPC axon terminals abrogated the CNO‐induced spatial learning (C) and memory (D–F) improvement; (G) The swimming speed was not affected (*p* > 0.05). (H) The trajectory maps on BM. (I–K) BM data show that microinjection of intra‐mPFC GluR antagonists into mice expressing hM3Dq in vHPC axon terminals blocked the CNO‐induced spatial learning (I) and memory (J, K) improvement (*n* = 6/group, one‐way analysis of variance (ANOVA) and Tukey's multiple comparisons test; * NBQX versus saline, # D‐AP5 versus saline; *p < 0.05, **p < 0.01, ***p < 0.001, ****p < 0.0001, # *p* < 0.05, ## *p* < 0.01). (L) No differences were found in total distance traveled (*p* > 0.05).

To determine whether the changes in the observed memory‐related behaviors were mediated by glutamatergic inputs from the HPC to the PFC, we conducted an additional series of trials (Figure 5). First, to selectively express hM3Dq in vHPC projection neurons, we injected 0.5 μl of pAAV‐CaMKII*α*‐hM3Dq‐mCherry into the vHPC bilaterally (AP ‐3.1 mm, ML ± 3.0 mm, DV ‐3.9 mm) as described before and implanted a pair of guide cannulas in mPFC (AP + 1.8 mm, ML ± 0.4 mm, DV ‐1.4 mm). Next, 0.5 μl of glutamate receptor antagonists or saline was injected bilaterally via infusion cannulas 30 min prior to CNO injection (Figure 5A). The mice in the antagonist groups (DAP5 and NBQX) performed significantly worse in the MWM and BM tests of spatial learning and memory function compared with mice in the Saline group. All mice in the MWM test were trained for 5 days to find the hidden platform. Spatial navigation was accomplished in the Saline group, demonstrating that the antagonist groups had learning deficits (Figure 5C). On day 6, we removed the hidden platform and found that the number of platform crossings and the duration in the target quadrant were significantly decreased, and the primary latency was significantly prolonged in the antagonist groups (Figure 5D–F). The locomotion was similar among the groups (Figure 5G). In the BM test, all mice were trained for 5 days to explore the target hole. On day 6, we removed the target hole and found that the antagonist groups of mice spent longer searching for the target hole and made fewer visits to the target hole compared with the Saline group of mice (Figure 5H–K). The movement among groups was not affected (Figure 5M). These data indicate that glutamate receptors mediated the effects of HPC‐PFC pathway activation on CLP‐induced cognitive dysfunction.

**FIGURE 6:**
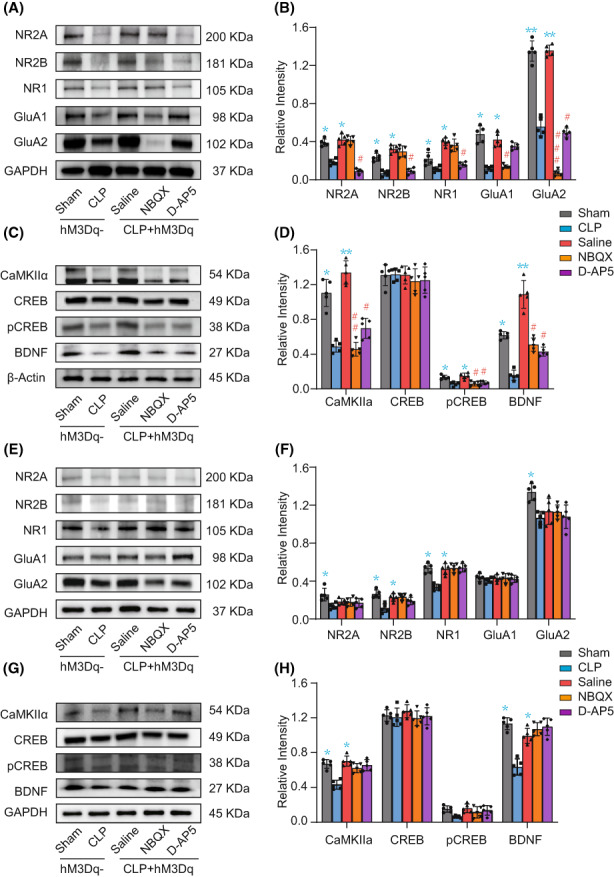
The protective mechanism of the HPC‐PFC pathway in CLP‐induced cognitive dysfunction may be mediated via activation of the glutamate receptor signaling pathway. (A, B) CLP reduced levels of AMDAR (GluA1, GluA2) and NMDAR (NR1, NR2A, NR2B) in PFC, while activation of HPC‐PFC increased the levels. Treatment with the AMDAR inhibitor (NBQX) and NMDAR inhibitor (D‐AP5) effectively reduced the expression of AMDAR and NMDAR in PFC, respectively. (C, D) CLP reduced the levels of CaMKIIα, pCREB, and BDNF in PFC, while activation of HPC‐mPFC increased the expression. The level of total CREB protein remained unchanged. NBQX and D‐AP5 effectively reduced the expression of CaMKIIα, pCREB and BDNF in PFC. (E, F) CLP reduced the levels of NMDAR (NR2A/B, NR1) and GluA2 in HPC, while the activation of HPC‐PFC increased the levels of NR2B and NR1. The AMDAR inhibitor (NBQX) and NMDAR inhibitor (D‐AP5) did not inhibit the expression of AMDAR and NMDAR in HPC. (G, H) CLP reduced the levels of CaMKIIα and BDNF in HPC, while activation of HPC‐mPFC increased the expression. The levels of total CREB and pCREB proteins remained unchanged. NBQX and D‐AP5 did not inhibit the expression of CaMKIIα, pCREB and BDNF in HPC. (*n* = 5/group, one‐way analysis of variance (ANOVA) with Bonferroni's multiple comparisons test, * versus CLP, # versus saline, *p < 0.05, **p < 0.01, # *p* < 0.05, ## *p* < 0.01, ### *p* < 0.001).

To further explore the molecular mechanisms of regulation of cognitive dysfunction mediated via the HPC‐PFC pathway in CLP‐induced SAE, we used western blotting analysis to analyze the expression of AMPAR (GluA1, GluA2), NMDAR (NR1, NR2A/2B), and downstream signaling molecules including CaMKIIa, pCREB/CREB, and BDNF in PFC (Figure 6A–D) and HPC (Figure 6E, F). The results showed that the levels of NMDAR (NR1, NR2A/2B), AMPAR (GluA1, GluA2), and the downstream signaling molecules including CaMKIIa, pCREB, and BDNF in PFC increased significantly after activation of the HPC‐PFC pathway, and that inhibiting glutamate receptors via NBQX or D‐AP5 abrogated this increase (Figure 6A, B). NBQX and D‐AP5 also inhibited the expression of downstream effectors, including CaMKIIa, pCREB, and BDNF, with no obvious change in CREB (Figure 6C, D). CLP reduced the levels of NR1, NR2A/B and GluA2 in HPC, while the activation of HPC‐mPFC simply increased the levels of NR1 and NR2B. Inhibitors of AMDAR (NBQX) and NMDAR (D‐AP5) did not inhibit the expression of these receptors in HPC (Figure 6E, F). CLP reduced the levels of CaMKIIα and BDNF in HPC, while the activation of HPC‐mPFC increased their expression. The levels of total CREB and pCREB proteins were not changed. NBQX and D‐AP5 did not inhibit the expression of CaMKIIα, pCREB and BDNF in HPC (Figure 6G, H). These data suggest that the glutamate receptor‐mediated CaMKII/CREB/BDNF signaling pathway modulated the HPC‐mPFC pathway in CLP‐induced cognitive dysfunction.

## 4 DISCUSSION

Chemogenetic techniques and applications have become increasingly prevalent in the field of neuroscience. They are routinely used to identify neurocircuits related to emotional and cognitive function in animals.^21^ In this study, we used DREADDs based on modified G‐coupled protein receptors that are activated by CNO to investigate the role of the HPC‐PFC pathway in cognitive dysfunction of mice showing CLP‐induced sepsis. We successfully validated the projection pathway from HPC to PFC and found that the HPC‐PFC pathway was inhibited in CLP‐induced sepsis. Further, chemogenetic activation of the HPC‐PFC pathway ameliorated the cognitive dysfunction of mice with sepsis. These findings suggest that HPC‐PFC inputs may be one of the central pathways involved in SAE pathogenesis. Further, the effect of HPC‐PFC in improving cognitive function was blocked by glutamate receptor (AMPAR or NMDAR) antagonists. Activation of the HPC‐mPFC pathway increased the levels of postsynaptic glutamate receptors and the downstream signaling molecules including CaMKII, pCREB, and BDNF in the CLP model of SAE. Therefore, we speculate that the impairment of the HPC‐PFC pathway mediated by glutamate receptors may be one of the mechanisms underlying cognitive dysfunction in SAE. To the best of our knowledge, this is the first demonstration of the role of the HPC‐PFC pathway in sepsis‐related cognitive dysfunction, thus providing new insights into SAE pathogenesis.

Both CLP and LPS models of sepsis are the most frequently used to evaluate cognitive dysfunction.^22^ CLP affects cognitive function via microglial activation and cerebral inflammation. Compared with LPS‐induced sepsis, the CLP model induces slower but concomitant increase in inflammatory factors similar to human sepsis.^23^ Thus, cognitive dysfunction occurs subsequent to CLP induction. Chavan et al.^24^ reported that deficits in spatial memory persisted for at least 4 months after CLP, suggesting the reliability and application of the model in assessing long‐term cognitive function in SAE. The CLP model is currently considered the gold standard in the study of sepsis.^18^ However, the CLP model indicates a large variation in mortality and may not be appropriate for some experiments. The length of cecal ligation was the most predominant factor affecting survival of the mouse model beyond needle size and puncture times.^25^ To establish an appropriate and reproducible animal model of SAE, we ligatured uniformly at 1 cm from the cecal apex, which resulted in a 40% mortality rate (Figure 1B). Thus, the model facilitates investigation of long‐term cognitive and memory dysfunction. Consistent with previous results, our CLP models exhibited serious cognitive dysfunction in MWM and BM tests 7 days after CLP onset.

The pathogenesis of sepsis‐induced cognitive impairment may be complex. Numerous drugs or chemical components have been used to ameliorate sepsis‐induced cognitive dysfunction and neuroinflammation in mice. These include traditional Chinese medicine,^26^ acetylcholinesterase inhibitors,^27^ angiotensin‐converting enzyme (ACE) inhibitors,^28^ the benzodiazepine agonist MRK‐016,^29^ the NMDA antagonist MK‐801,^30^ ketamine,^31^ morphine,^32^ morin,^33^ and others. Among these strategies, SAE prevention and treatment is achieved mainly via anti‐inflammatory and antioxidative interventions which indicates the importance of inflammatory pathways and oxidative stress in SAE pathogenesis. Nonpharmacological interventions including physical exercise^34^ and environmental rehabilitation^35^ have also been recently investigated to alleviate sepsis‐induced cognitive impairment. In contrast, the neurological pathways associated with SAE are seldom reported. The HPC‐PFC pathway is thought to be associated with memory^7^, ^36^ and anxiety regulation.^37^ Further, the HPC‐PFC pathway plays a crucial role in encoding spatial memory.^7^ In this study, we detected alterations in spatial learning and memory. By selectively activating the HPC‐PFC pathway via chemogenetic strategies, we found that the HPC‐PFC pathway regulates spatial learning and memory in the CLP model of sepsis. However, further experimental studies are needed to corroborate the preliminary findings reported here.

The results of our present study also showed that the activation of HPC‐PFC modulated the signaling pathways in the PFC, including those of AMPAR, NMDAR, CaMKII, CREB and BDNF, and collectively improved the working memory of septic mice in MWM and BM tests. Glutamate receptors (GluRs) mediate the majority of excitatory neurotransmission in the central nervous system (CNS). NMDAR and AMPAR are ionotropic GluRs and play a vital role in synaptic plasticity and synaptic transmission.^38^ NMDAR‐dependent plasticity is important for episodic‐like memory. Further, the activation of NMDA receptors triggers memory formation.^39^ AMPAR ensures information transfer via NMDAR‐dependent synaptic plasticity, specifically, by activating voltage‐dependent calcium channels, resulting in transient influx of Ca^2+^, and enhanced excitatory synaptic transmission.^40^ Calcium influx leads to the activation of CaMKII, which induces long‐term potentiation (LTP) and improves learning and memory.^41^ CaMKII promotes CREB phosphorylation (p‐CREB),^42^ which results in BDNF transcription.^43^ As an important molecule involved in memory, BDNF promotes excitatory signaling and LTP.^44^ In the current study, we analyzed the expression of AMPAR and NMDAR, and their downstream signaling molecules, including CaMKII, CREB, pCREB, and BDNF in the PFC. Our data showed that the effect of HPC‐PFC in improving cognitive dysfunction could be blocked by antagonists of both the GluRs (AMPAR or DMDAR). The levels of GluRs and CaMKII/pCREB/BDNF were reduced in septic mice but increased after targeted HPC‐PFC activation in PFC. However, no changes in the expression of GluRs and their downstream signaling molecules in HPC were found after the injection of antagonists. These results indicate that the GluR‐mediated CaMKII/CREB/BDNF signaling pathway plays a role in the HPC‐PFC pathway during CLP‐induced cognitive dysfunction.

The study has a few important limitations. First, we did not detect specific biomarkers of neuronal injury in SAE. Second, the behavioral manifestations of mice were only considered 714 days after CLP, and long‐term effects have yet to be determined. In addition, some studies revealed sex differences in gene expression^45^ and mitochondrial and related proteins^46^ in rat brain microvessels. There are also reported sex differences regarding brain metabolism^47^ and cerebral perfusion‐trajectories^48^ in human brain. Sex differences may impact the results. Given that all of our experiments were performed in male mice but not in female mice, our findings may not be generalizable. Finally, though the GluR‐mediated CaMKII/CREB/BDNF signaling pathway was associated with the HPC‐PFC pathway in SAE, it does not prove causality.

In conclusion, we identified a prominent excitatory pathway from HPC to PFC and revealed the important role of the HPC‐PFC pathway in improving cognitive dysfunction in mice with CLP‐induced sepsis. Our study may provide a novel pathogenetic mechanism and a potential new treatment direction for SAE.

## AUTHOR CONTRIBUTION

Qian‐Yi Peng designed the research. Yu Zou and Wei Chen collected the data. Cheng‐long Ge performed the data analysis and wrote the manuscript. Li‐Na Zhang and Yu‐Hang Ai reviewed the manuscript. All the authors reviewed and approved the manuscript.

## CONFLICT OF INTEREST

The authors declare that there is no conflict of interest.

## Data Availability

All processed data used in this study can be obtained from the corresponding author on reasonable request.
